# The effect of Central and peripheral thyroid resistance indices on diabetic retinopathy: a study of hospitalized euthyroid patients with T2DM in China

**DOI:** 10.1080/07853890.2023.2249017

**Published:** 2023-08-26

**Authors:** Xin Zhao, Jianbin Sun, Xiumei Xu, Sixu Xin, Xiaomei Zhang

**Affiliations:** Department of Endocrinology, Peking University International Hospital, Beijing, P.R. China

**Keywords:** Type 2 diabetes mellitus, thyroid hormone sensitivity, glycosylated hemoglobin, diabetic retinopathy

## Abstract

**Objective:**

This study aims to explore the correlation between central and peripheral thyroid resistance indices and diabetic retinopathy(DR) in patients with type 2 diabetes mellitus (T2DM), so as to provide a clinical basis for the prevention and treatment of diabetic retinopathy.

**Methods:**

This study retrospectively analyzed 1249 euthyroid patients with T2DM hospitalized in the Department of Endocrinology, Peking University International Hospital from January 2017 to June 2022, including 852 males and 397 females, with an average age of 54.73 ± 13.40 years. According to the degree of DR, the patients were divided into three groups including the no diabetic retinopathy (NDR) group, non-proliferative diabetic retinopathy (NPDR) group and proliferative diabetic retinopathy (PDR) group.

**Results:**

Free thymidine (FT4), thyroid stimulating hormone (TSH), thyroid feedback quantile index (TFQI), thyrotropin-T4 resistance index (TT4RI), thyroid stimulating hormone index (TSHI) and free triiodothyronine/free thyroxine (FT3/FT4) levels among the three groups were significantly different, with the NDR group having lowest TSH, TFQI, TT4QI, TSHI and the highest in the PDR group (all *p* < 0.05). Logistic regression showed that after adjusting for age, body mass index (BMI), sex, diabetes duration, blood pressure, blood lipid, HbA1c, lower level of FT4 was an independent risk factor for DR, high level of TSH, TFQI, TSHI and TT4RI were independent risk factors for DR. Central and peripheral thyroid sensitivity indices have predictive value for DR, the overall predictive accuracy of FT3/FT4 was 0.61 (95%CI 0.57, 0.65), the overall predictive accuracy of TFQI was 0.66(95%CI 0.63, 0.70), the overall predictive accuracy of TSHI was 0.66(95%CI 0.62, 0.68), the overall predictive accuracy of TT4RI was 0.63 (95%CI 0.59, 0.66).

**Conclusion:**

The reduction of central and peripheral thyroid hormone sensitivity is an independent risk factor for DR. These results can help predict the risk of the occurrence and development of DR, which may provide a clinical basis for the prevention and treatment of DR in T2DM patients.

## 1. Introduction

With the rapid development of people’s material living standards, there has been a notable increase in the prevalence of diabetes mellitus (DM) [1]. In 2019, the global population of individuals with diabetes reached 463 million. Projections indicate that this figure will rise to nearly 580 million by 2023 and further escalate to 700 million by 2045 [2]. Diabetic retinopathy (DR) stands as a prominent contributor to visual impairment and blindness. During the initial stage, patients with DR show no noticeable clinical symptoms. As the disease advances, it can be categorized into two stages: the non-proliferative stage and the proliferative stage. Prolonged hyperglycemia and delayed treatment can lead to microangiopathy, hard exudates, cotton wool spots or soft exudates, neovascularization, vitreous haemorrhage, fibrous hyperplasia, and loss of vision due to retinal detachment [3].

Previous studies have established a clear connection between DM and its associated complications with thyroid hormones (THs). Compared with healthy people, the prevalence of thyroid disease (TD), especially hypothyroidism, in DM patients is higher [4]. Currently, there exist conflicting findings regarding the relationship between THs and DR. One study has demonstrated a negative correlation between the level of free triiodothyronine (FT3) within the normal range and DR in euthyroid patients with type 2 diabetes mellitus (T2DM). However, no significant associations were observed between free thyroxine (FT4), thyroid-stimulating hormones (TSHs), and DR in the same patient population [5]. Another study pointed out that there was no significant correlation between the TSH level and DR [6]. Contrarily, a study has indicated that among patients with T2DM, there was a higher incidence of retinopathy among those with elevated TSH levels [7]. Currently, the precise relationship and underlying mechanisms linking THs and DR remain uncertain. Impaired TH sensitivity can be divided into central sensitivity impairment and peripheral sensitivity impairment. Central sensitivity impairment refers to a disruption in the feedback circuit of the hypothalamus-pituitary-thyroid (HPT) axis within the central system, which can occur due to factors like prolonged fasting or certain disease conditions [8]. Peripheral sensitivity impairment, on the other hand, is characterized by a reduction in the metabolism regulated by THs. At present, the commonly used indices to reflect TH sensitivity include the thyrotropin-T4 resistance index (TT4RI), thyroid stimulating hormone index (TSHI), thyroid feedback quantile index (TFQI), and the ratio of free triiodothyronine/free thyroxine (FT3/FT4). TSHI, TT4RI, and TFQI may reflect alterations in the sensitivity of thyroid hormones to the HPT axis [9,10], and the FT3/FT4 ratio may reflect the sensitivity of peripheral tissues to changes in TH levels.

Currently, there is limited research both domestically and internationally regarding the correlation between impaired TH sensitivity and diabetic complications. Particularly, there is a lack of studies investigating the connection between impaired TH sensitivity and DR. Therefore, this study aims to assess the relationship between THs, central and peripheral TH sensitivity, and DR among euthyroid patients with T2DM. The findings of this research endeavour will offer valuable clinical insights into the prevention and management of DR.

## 2. Research subjects and method

### 2.1. Ethics statement

The study was approved by the Ethics Committee of Peking University International Hospital and was conducted in accordance with the ethics standards of institutional and national research committees and the 1964 Helsinki Declaration and its later amendments or comparable ethics standards. As the study involved the retrospective analysis of clinical data, the requirement for written informed consent was waived. The number of the ethics approval is 2022-KY-0030-01.

### 2.2. Research subjects

This study retrospectively analyzed 1249 T2DM patients hospitalized in the Endocrinology Department of Peking University International Hospital from January 2017 to June 2022, including 852 males and 397 females. All patients were accorded the diagnostic criteria for diabetes of the World Health Organization (WHO) in 1999 [11], including (1) typical symptoms of diabetes and random blood glucose ≥11.1 mmol/l; (2) fasting blood glucose ≥7.0 mmol/l; (3) in OGTT test, blood glucose ≥11.1 mmol/l after taking 75 g glucose for 2 h. If one of the three criteria is still met, it will be diagnosed as diabetes, and it will meet the diagnostic criteria of T2DM according to clinical classification. Exclusion criteria of this study: (1) patients with gestational diabetes mellitus, type 1 diabetes mellitus or other special types of diabetes; (2) acute complications of diabetes mellitus; (3) patients with thyroid disease history (history of thyroid dysfunction, history of thyroidectomy), with the application of thyroid drugs (levothyroxine or antithyroid drugs) or with parathyroid gland, adrenal gland disease, pituitary disease and other endocrine diseases; (4) patients with haematological diseases and malignant tumours.

### 2.3. Methods

#### 2.3.1. General conditions and clinical data

The data of 1249 patients were retrospectively analyzed, and the general conditions of the patients, including sex, age, height, weight, diabetes duration, systolic blood pressure (SBP), diastolic blood pressure (DBP) and hypoglycemic medications were all recorded. The body mass index (BMI) was calculated using the formula: BMI (kg/m^2^) =weight (kg)/body height^2^ (m^2^).

#### 2.3.2. Laboratory biochemical indices

All subjects were fasting for more than 8 h, and venous blood was drawn in the morning of the next day. The biochemical indices were tested including glycosylated haemoglobin (HbA1c), fasting blood glucose (FBG), postprandial blood glucose (PBG), triglyceride (TG), total cholesterol (TC), high-density lipoprotein cholesterol (HDL-C), low-density lipoprotein cholesterol (LDL-C), serum creatinine (sCr), uric acid (UA), free thymidine (FT4) and free triiodothyronine (FT3) and TSH (thyroid stimulating hormone). HbA1c is determined by high-performance liquid chromatography.

The assessment index of THs sensitivity is as follows:

(1) TFQI Index:

TFQI=CDFFT4-(1-CDFTSH); First rank the FT4 and TSH from the minimum to the maximum. Then, according to the principle of empirical cumulative distribution function (CDF), transform the probability distribution of FT4 and TSH into the probability quantile between 0 and 1. After calculating, the range of TFQI is from − 1 to 1. TFQI is negative, indicating that HPT axis is more sensitive to the change of FT4; a positive value indicates that the HPT axis is relatively insensitive to the change of FT4; 0 indicates that the sensitivity of HPT axis to FT4 is normal.

(2) TT4RI: TT4RI was calculated as the formula: TT4RI=FT4×TSH.

(3) TSHI: TSHI was calculated as the formula: TSHI = LnTSH + 0.1345 × FT4.

The higher the TSHI and TT4RI values, the lower the central sensitivity to THs.

(4) FT3/FT4: the higher the FT3/FT4 value, the higher the peripheral sensitivity to TH.

#### 2.3.3. Diabetic retinoscopy

All subjects were examined for DR. According to the results, the patients were divided into three groups: no DR (NDR) group, non-proliferative retinopathy (NPDR) group and proliferative retinopathy (PDR) group. The criteria are as follows: NPDR includes the first stage in which small bleeding spots or micro angiomas are found, the second stage in which hard exudation was found and the third stage in which soft cotton sample was found. PDR includes the fourth in which blood accumulation and neovascularization of the retinal vitreous body were found, the fifth stage in which fibrovascular hyperplasia and subsequent vitreous tissue were found and the sixth stage in which traction retinal detachment leads to blindness.

#### 2.3.4. Statistical analysis

Statistical analysis was performed with the SPSS Version 22.0 software (IBM, Chicago, IL, USA). The data were analyzed using the Kolmogorov-Smirnov test, and all variables had a normal distribution and were expressed as the mean ± standard deviation. Multi-group comparisons of the data were compared with the one-way analysis of variance (ANOVA). The statistical description of counting data is based on constituent ratio or rate and comparisons among groups were made using χ2 test. Unconditional logistic regression models were used for univariate and multivariate analysis of the factors, and OR and 95%CI were calculated. The receiver operating characteristic (ROC) curves were plotted and the area under the ROC curve (AUC) was calculated. All statistical tests were performed with two-sided tests, and *p* < 0.05 was considered to be statistically significant.

## 3. Results

### 3.1. Comparison of general conditions and biochemical indices among the three groups

The patients in NDR group were the youngest and had the shortest diabetes duration among the three groups (all *p* < 0.05). The UA, UACR and eGFR levels among the three groups were significantly different, with the PDR group having the highest UACR and lowest eGFR levels (all *p* < 0.05). The HbA1c, FBG and PBG levels among the three groups were significantly different, with NDR group having the lowest HbA1c, FBG and PBG among the three groups (all *p* < 0.05). In addition, the FT4, TSH, TFQI, TT4QI, TSHI and FT3/FT4 levels among the three groups were significantly different, with the NDR group having lowest TSH, TFQI, TT4QI, TSHI and the highest in the PDR group (all *p* < 0.05). The SBP, DBP, TC, TG, LDL-C, HDL-C, FT3 were not significantly different among the three groups (all *p* < 0.05). There was no significant statistical difference in hypoglycemic drugs among the three groups (all *p* < 0.05) (Table 1).

**Table 1.** Comparison of general conditions and biochemical indices among three groups.

	NDR	NPDR	PDR		
Index	(*n* = 892)	(*n* = 227)	(*n* = 130)	F(*X*^2^)	*P*
Age (years)	54.01 ± 13.26	56.63 ± 13.07^a^	56.38 ± 14.51^a^	4.59	<0.05
Sex (male%)	614(68.83%)	143(63.00%)	95(73.08%)	4.42	0.11
BMI (kg/m^2^)	26.11 ± 3.92	25.76 ± 3.57	26.07 ± 4.10	0.68	0.51
Duration(years)	8.35 ± 7.31	12.25 ± 7.65^a^	12.19 ± 8.22^a^	34.36	<0.05
SBP (mmHg)	133.10 ± 35.45	136.77 ± 70.94	135.75 ± 20.42	0.77	0.46
DBP (mmHg)	79.71 ± 11.20	79.14 ± 10.92	80.61 ± 12.55	0.70	0.50
TC (mmol/L)	4.42 ± 1.21	4.33 ± 1.28	4.35 ± 1.27	0.47	0.62
TG (mmol/L)	2.16 ± 1.72	2.18 ± 2.34	2.26 ± 1.87	0.17	0.84
LDL-C(mmol/L)	2.62 ± 0.98	2.56 ± 1.09	2.53 ± 0.96	0.64	0.53
HDL-C(mmol/L)	1.00 ± 0.31	1.04 ± 0.33	0.98 ± 0.24	1.49	0.23
UA (umol/L)	349.01 ± 90.68	345.89 ± 100.66	386.07 ± 107.33^a,b^	8.85	<0.05
eGFR	99.10 ± 19.03	96.18 ± 20.82	91.14 ± 26.87^a,b^	9.11	<0.05
UACR (mg/g)	41.92 ± 188.31	108.36 ± 357.52^a^	294.48 ± 868.48^a,b^	28.94	<0.05
HbA1c (%)	8.66 ± 2.00	9.03 ± 1.98^a^	8.87 ± 1.94	2.99	<0.05
FBG (mmol/L)	8.91 ± 3.27	9.56 ± 3.89^a^	9.40 ± 3.63	3.65	<0.05
PBG (mmol/L)	12.53 ± 4.47	13.47 ± 4.46^a^	12.64 ± 4.53	3.69	<0.05
FT4 (pmol/l)	17.64 ± 2.08	16.96 ± 2.07^a^	16.47 ± 1.99^a,b^	21.74	<0.05
FT3 (pmol/l)	4.62 ± 2.36	4.39 ± 0.77	4.38 ± 0.72	1.65	0.19
TSH (uIU/ml)	1.86 ± 0.84	2.02 ± 0.85^a^	2.36 ± 0.96^a,b^	21.10	<0.05
TFQI	−0.05 ± 0.38	0.07 ± 0.36^a^	0.26 ± 0.41^a,b^	42.38	<0.05
TT4RI	30.36 ± 13.57	34.06 ± 14.51^a^	41.65 ± 17.86^a,b^	38.03	<0.05
TSHI	2.72 ± 0.54	2.89 ± 0.47^a^	3.14 ± 0.53^a,b^	40.35	<0.05
FT3/FT4	0.28 ± 0.15	0.26 ± 0.05^a^	0.25 ± 0.04^a^	5.28	<0.05
Medications					
Metformin	740(82.96%)	181(79.74%)	107(82.31%)	1.21	0.30
Sulfonylureas	322(36.10%)	82(36.12%)	46(35.38%)	1.43	0.29
Thiazolidinediones	12(1.35%)	5(2.20%)	7(5.38%)	1.89	0.11
Incretin mimetics	478 (53.59%)	119(52.42%)	77(59.23%)	0.87	0.45
Insulin	585(65.58%)	131(57.71%)	92(70.77%)	1.31	0.38

^a^compared with the NDR group, the difference was statistically significant (*p* < 0.05).

^b^compared with the NPDR, the difference was statistically significant (*p* < 0.05). BMI is for body mass index, SBP is systolic blood pressure, DBP is for diastolic blood pressure, FBG is for fasting blood glucose, PBG is for postprandial blood glucose, HbA1c is for glycosylated haemoglobin, eGFR is for glomerular filtration rates, UA is for uric acid, UACR is for urinary albuminuria creatinine ratio, TC is for total cholesterol, TG is for triglycerides, LDL-C is for low-density lipoprotein cholesterol, HDL-C is for high-density lipoprotein cholesterol, FT4 is for free thyroxine, FT3 is for free triiodothyronine, TSH is for thyroid-stimulating hormone, TT4RI is for thyrotroph thyroxine resistance index, TSHI is for thyroid-stimulating hormone index, TFQI is for thyroid feedback quantile -based index.

### 3.2. Logistic regression analysis of THs and sensitivity to THs and DR

Taking DR as a dependent variable and THs as independent variables, a univariate logistic regression model was established. The results showed that after adjusting for age, BMI, sex, diabetes duration, blood pressure, blood lipid and HbA1c, the indices including TSH, TFQI, TSHI and TT4RI were independent risk factors for DR (Table 2).

**Table 2.** Logistic regression analysis of sensitivity to THs and DR.

Index	Crude OR	95%CI	*p*	Adjust OR	95%CI	*p*
Thyroid hormone						
FT3(pmol/L)						
Low (2.40–4.10)	1			1		
Medium (4.11–4.79)	0.80	0.59,1.08	0.14	0.85	0.58,1.23	0.39
High (4.80–6.40)	0.86	0.64,1.17	0.33	1.28	0.85,1.92	0.23
FT4(pmol/L)						
Low (12.00–15.79)	1			1		
Medium (15.80–17.59)	0.59	0.43,0.81	<0.05	0.50	0.34,0.73	<0.05
High (17.60–22.00)	0.44	0.31,0.57	<0.05	0.36	0.25,0.53	<0.05
TSH (uIU/ml)						
Low (0.27–1.44)	1			1		
Medium (1.45–2.23)	1.27	0.93,1.75	0.13	1.14	0.78,1.66	0.51
High (2.24–4.20)	1.95	1.43,2.64	<0.05	1.94	1.35,2.80	<0.05
Peripheral thyroid resistance index						
FT3/FT4						
Low (0.12–0.24)	1			1		
Medium (0.25–0.29)	0.62	0.47,0.83	<0.05	0.69	0.48,0.97	<0.05
High (0.30–0.50)	0.41	0.29,0.57	<0.05	0.48	0.31,0.73	<0.05
Central thyroid resistance index						
TFQI						
Low (–0.96– −0.19)	1			1		
Medium (–0.18–0.18)	1.70	1.23,2.34	<0.05	1.50	1.03,2.19	<0.05
High (0.19–0.97)	2.36	1.72,3.24	<0.05	2.47	1.70,3.60	<0.05
TT4RI						
Low (4.10–23.87)	1			1		
Medium (23.88–37.04)	1.33	0.97,1.82	0.08	1.19	0.82,1.74	0.36
High (37.05–84.14)	1.98	1.46,2.69	<0.05	1.98	1.37,2.87	<0.05
TSHI						
Low (0.74–2.60)	1			1		
Medium (2.61–3.06)	1.52	1.11,2.10	<0.05	1.49	1.02,2.17	<0.05
High (3.07–4.24)	2.19	1.46,2.69	<0.05	2.42	1.66,3.52	<0.05

FT4 is for free thyroxine, FT3 is for free triiodothyronine, TSH is for thyroid-stimulating hormone, TT4RI is for thyrotroph thyroxine resistance index, TSHI is for thyroid-stimulating hormone index, TFQI is for thyroid feedback quantile -based index.

### 3.3. Logistic regression analysis of THs and the sensitivity to THs and PDR

Taking PDR as the dependent variable and THs as the independent variables, a univariate logistic regression model was established. The results showed that after adjusting for age, BMI, sex, duration, blood pressure, blood lipid and HbA1c, the indices including TSH, TFQI, TSHI and TT4RI were independent risk factors for PDR (Table 3).

**Table 3.** Logistic regression analysis of THs and sensitivity to THs and PDR.

Index	Crude OR	95%CI	*P*	Adjust OR	95%CI	*p*
Thyroid hormone						
FT3(pmol/L)						
Low (2.40–4.10)	1			1		
Medium (4.11–4.70)	0.80	0.48,1.36	0.41	0.57	0.28,1.14	0.11
High (4.71–6.40)	0.84	0.49,1.42	0.51	0.66	0.31,1.39	0.28
FT4(pmol/L)						
Low (12.00–15.79)	1			1		
Medium (15.80–17.59)	0.69	0.78,2.69	0.24	0.53	0.85,4.22	0.12
High (17.60–22.00)	0.43	0.24,0.77	<0.05	0.29	0.13,0.63	<0.05
TSH (uIU/ml)						
Low (0.27–1.44)	1			1		
Medium (1.45–2.23)	0.79	0.44,1.45	0.45	0.74	0.35,1.56	0.43
High (2.24–4.20)	1.79	1.04,3.07	<0.05	2.04	1.04,4.00	<0.05
Peripheral thyroid resistance index						
FT3/FT4						
Low (0.12–0.24)	1			1		
Medium (0.25–0.29)	0.67	0.42,1.09	0.10	0.63	0.33,1.20	0.16
High (0.30–0.40)	0.57	0.30,1.06	0.07	0.38	0.16,0.87	<0.05
Central thyroid resistance index						
TFQI						
Low (–0.72– −0.19)	1			1		
Medium (–0.18–0.18)	0.92	0.50,1.69	0.78	1.14	0.56,2.36	0.71
High (0.19–0.97)	1.88	1.07,3.33	<0.05	2.33	1.17,4.65	<0.05
TT4RI						
Low (9.84–23.87)	1			1		
Medium (23.88–36.89)	0.90	0.49,1.65	0.73	1.06	0.49,2.28	0.88
High (36.90–84.14)	2.22	1.28,3.86	<0.05	2.66	1.29,5.47	<0.05
TSHI						
Low (2.01–2.60)	1			1		
Medium (2.61–3.06)	1.24	0.67,2.29	0.50	1.49	0.72,3.10	0.28
High (3.07–4.24)	2.44	1.37,4.33	<0.05	2.97	1.46,6.03	<0.05

FT4 is for free thyroxine, FT3 is for free triiodothyronine, TSH is for thyroid-stimulating hormone, TT4RI is for thyrotroph thyroxine resistance index, TSHI is for thyroid-stimulating hormone index, TFQI is for thyroid feedback quantile-based index.

### 3.4. Univariate predictive model of DR with THs and sensitivity to THs

**Figure 1.**
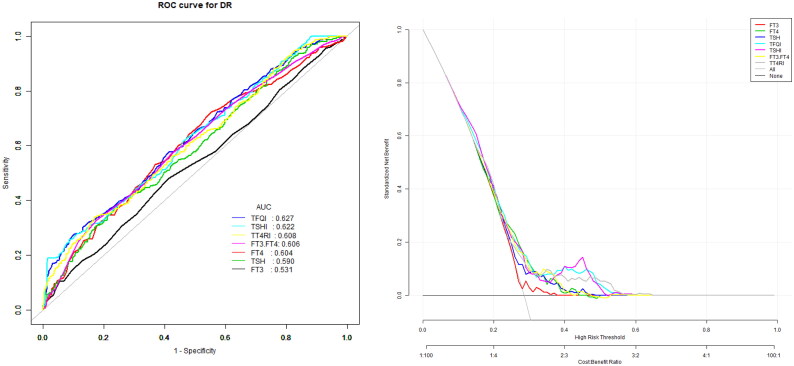
The overall predictive accuracy of FT3/FT4 for DR was 0.61 (95%CI 0.57,0.65), the overall predictive accuracy of FT4 for DR was 0.59 (95%CI 0.55, 0.63), the overall predictive accuracy of FT3 of DR was 0.55 (95%CI 0.51, 0.60), the overall predictive accuracy of TSH for DR was 0.62 (95%CI 0.58, 0.65), the overall predictive accuracy of TFQI of DR was 0.66 (95%CI 0.63, 0.70), the overall predictive accuracy of TSHI for DR was 0.66 (95%CI 0.62, 0.68), the overall predictive accuracy of TT4RI of DR was 0.63 (95%CI 0.59, 0.66).

The model for predicting the risk of DR with variables including THs and THs sensitivity indices showed that the AUC of the models ranked TSHI (0.66) = TFQI (0.66) > TT4RI (0.63) > TSH (0.62) > FT3/FT4 (0.61) > FT4 (0.59) > FT3 (0.55). The corresponding cut-off points of FT4, FT3, TSH are 16.95 pmol/L, 4.35 pmol/L and 2.38uIU/ml respectively. The corresponding cut-off points of FT3/FT4, TFQI, TT4RI and TSHI are 0.24, 0.44, 44 and 3.57 (Table 4 and Figure 1).

**Table 4.** Univariate predictive model of DR.

Index	AUC(95%CI)	Specificity	Sensitivity	Cut-off
FT4	0.59(0.55, 0.63)	0.45	0.72	16.05
FT3	0.55(0.51, 0.60)	0.59	0.48	4.35
TSH	0.62(0.58, 0.65)	0.75	0.40	2.38
FT3/FT4	0.61(0.57, 0.65)	0.82	0.33	0.24
TFQI	0.66(0.63, 0.70)	0.90	0.27	0.44
TT4RI	0.63(0.59, 0.66)	0.83	0.34	44.00
TSHI	0.66(0.62, 0.68)	0.98	0.19	3.57

FT4 is for free thyroxine, FT3 is for free triiodothyronine, TSH is for thyroid-stimulating hormone, TT4RI is for thyrotroph thyroxine resistance index, TSHI is for thyroid-stimulating hormone index, TFQI is for thyroid feedback quantile -based index.

### 3.5. Univariate predictive model of PDR with THs and sensitivity to THs

**Figure 2.**
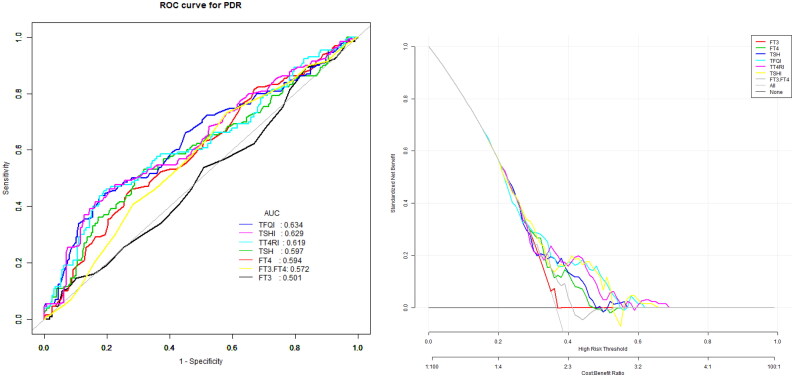
The overall predictive accuracy of FT3/FT4 for PDR was 0.54 (95%CI 0.48, 0.60), the overall predictive accuracy of FT4 for PDR was 0.58 (95%CI 0.53, 0.65), the overall predictive accuracy of FT3 of PDR was 0.51 (95%CI 0.45, 0.57), the overall predictive accuracy of TSH for PDR was 0.59 (95%CI 0.53, 0.65), the overall predictive accuracy of TFQI of PDR was 0.63 (95%CI 0.57, 0.69), the overall predictive accuracy of TSHI for PDR was 0.63 (95%CI 0.56, 0.69), the overall predictive accuracy of TT4RI of PDR was 0.62 (95%CI 0.56,0.68).

The model for predicting the risk of PDR with variables including THs and THs sensitivity index showed that the AUC of the models ranked TSHI (0.63) =TFQI (0.63) > TT4RI (0.62) > TSH (0.59) > FT4(0.58) > FT3/FT4 (0.54) > FT3 (0.51). The corresponding cut-off points of FT4, FT3, TSH are 18.05 pmol/L, 3.65 pmol/L and 2.36uIU/ml respectively. The corresponding cut-off points of FT3/FT4, TFQI, TT4RI and TSHI are 0.28 0.44,46.18 and 3.29 (Table 5 and Figure 2).

**Table 5.** Univariate predictive model of PDR.

Index	AUC(95%CI)	Specificity	Sensitivity	Cut-off
FT4	0.58(0.53, 0.65)	0.72	0.46	18.05
FT3	0.51(0.45, 0.57)	0.16	0.89	3.65
TSH	0.59(0.53, 0.65)	0.68	0.53	2.36
FT3/FT4	0.54(0.48, 0.60)	0.42	0.73	0.28
TFQI	0.63(0.57, 0.69)	0.82	0.44	0.44
TT4RI	0.62(0.56, 0.68)	0.81	0.45	46.18
TSHI	0.63(0.56, 0.69)	0.79	0.46	3.29

FT4 is for free thyroxine, FT3 is for free triiodothyronine, TSH is for thyroid-stimulating hormone, TT4RI is for thyrotroph thyroxine resistance index, TSHI is for thyroid-stimulating hormone index, TFQI is for thyroid feedback quantile -based index.

### 3.6. Multivariate predictive model for the risk of DR

**Figure 3.**
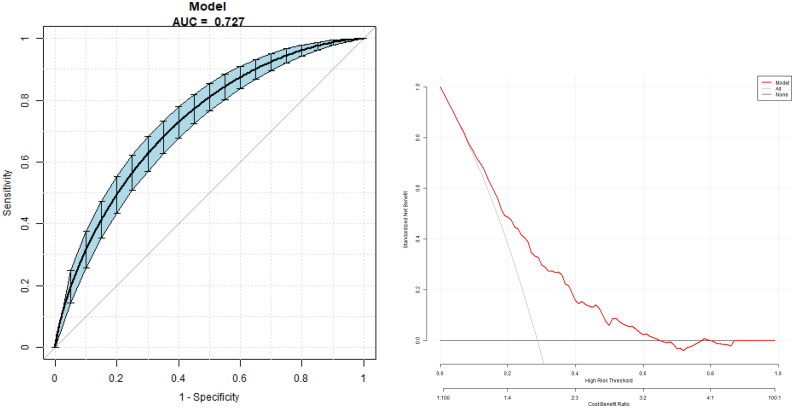
The overall predictive accuracy of a multivariate predictive model for the risk of DR. The AUC is 0.73 (95% CI 0.70, 0.76), with a specificity of 78.85%, a sensitivity of 55.74% and an accuracy of 72.21%.

The multivariate predictive model was established with DR as the dependent variable and age, duration, UA, Egfr, HbA1c, FT3/FT4, TFQI, TT4QI and TSHI as independent variables. The regression equation is logit (DR) = −3.23727 − 0.00934 × age +0.07810 × duration +0.00170*UA −0.00148 × eGFR +0.07341 × HbA1c + 0.38299*TFQI +0.00358 × TT4RI +0.69236 × TSHI −4.47422 × FT3/FT4, the AUC is 0.73 (95% CI 0.70, 0.76), a specificity of 78.85%, a sensitivity of 55.74 P% and an accuracy of 72.21% (Figure 3).

### 3.7. Multivariate predictive model for the risk of PDR

**Figure 4.**
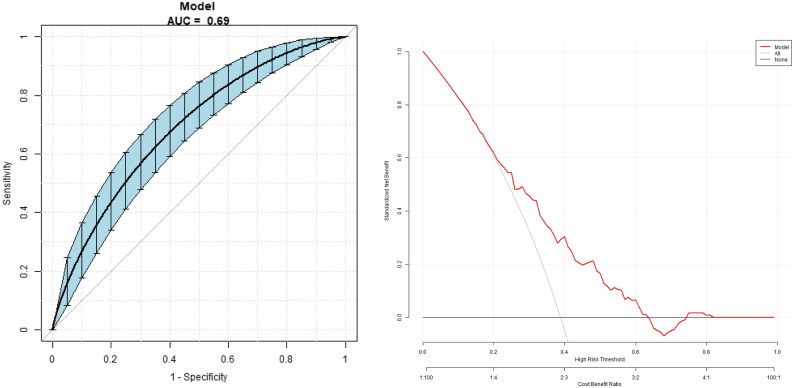
The overall predictive accuracy of a multivariate predictive model for the risk of PDR. The AUC is 0.69 (95% CI 0.63, 0.75), with a specificity of 51.93%, a sensitivity of 80.00% and an accuracy of 62.84%.

The multivariate predictive model was established with PDR as the dependent variable and age, duration, UA, eGFR, HbA1c, FT3/FT4, TFQI, TT4QI and TSHI as independent variables. The regression equation is logit (PDR) = 0.11446 − 0.00897*age +0.01095*duration +0.00306*UA −0.00649*eGFR −0.00028*HbA1c + 1.27457*TFQI +0.01356*TT4RI −0.27423*TSHI −2.32958*FT3/FT4, the AUC is 0.69 (95% CI 0.63, 0.75), a specificity of 51.93%, a sensitivity of 80.00% and an accuracy of 62.84% (Figure 4).

## 4. Discussion

THs increase energy consumption and heat production [12], increase glucose and fatty acid oxidation in the muscle [13] and liver [14], and at the same time can increase the decomposition of adipose tissues [15]. These actions can lead to weight reduction and significantly influence metabolic processes. In terms of glucose metabolism, THs can directly or indirectly regulate insulin secretion and glucose homeostasis by affecting glucose-induced insulin secretion or the sensitivity of islet B cells [16]. Researchers have discovered contrasting findings regarding the connection between diabetes or metabolic syndrome and TSHs as opposed to THs. Some studies suggested a correlation between diabetes or metabolic syndrome and TSHs while excluding any significant association with THs. Conversely, other studies indicate that diabetes or metabolic syndrome is exclusively linked to THs while downplaying the role of TSHs [17].

Several biological changes associated with TH levels have been found, substantiating the influence of THs on microvascular complications. These changes include the impact of THs on metabolic processes within the body, the development of chronic inflammation due to decreased TH levels, and the impairment of vascular endothelial function [18]. At present, there are limited studies on the correlation between THs and DR, and the results are quite controversial. Studies have found that there are significant differences in the prevalence of DR among patients with varying levels of FT3. However, no substantial disparity has been observed in the prevalence of DR across groups with varying levels of FT4, TSHs, and anti-thyroid peroxidase antibodies (TPOAbs) [5]. Contrasting findings from other studies indicate that after adjusting for other risk factors, there is a significant association between lower levels of FT4 within the normal range, higher levels of TSHs, and the prevalence of DR [19]. The variation in research findings could be attributed to the sample size of each study. Yonghui Hu [20] concluded that the serum FT3 and FT4 levels were negatively correlated with DR in euthyroid patients with T2DM, independent of traditional risk factors. Our research findings align with the aforementioned study, indicating a similarity in results. However, our study encompassed a significantly larger sample size of 1249 subjects, surpassing the sample size of previous studies. In this study, we found that among euthyroid patients, the high level of FT4 within the normal range is a protective factor against DR (OR = 0.44,95%CI 0.31, 0.57), and after adjusting for age, sex, duration, blood pressure, blood glucose, and other factors, the high level of FT4 remains an independent protective factor against the occurrence of DR (OR = 0.36,95%CI 0.25,0.53).

The correlation between TH sensitivity and retinopathy has been extensively studied using animal models. These studies have provided valuable insights into the underlying pathophysiological mechanisms linking THs and retinopathy. Kocaturka found that rats with hypothyroidism exhibited reduced levels of sirtuin 2 protein expression in the retinal ganglion cell layer. This finding suggests that the significant deficiency of THs adversely impacted the normal development of the retinal cell structure [21]. Several studies have investigated the relationship between TD and retinal blood flow. Ittermann observed that when compared to euthyroid patients, patients with higher levels of TSHs had lower arteriovenous indices and narrower retinal arteries. Microvascular injury caused by local hypertension and atherosclerosis can lead to arterial contraction [22]. In patients with DR, chronic inflammation can also damage the structure and function of the vascular endothelium. THs may influence retinal diseases through the following mechanisms: (1) In rat models of both type 1 and 2 diabetes, a higher number of dual cones was detectable, which was found to be associated with the levels of FT4. Postmortem human retinas also exhibited the presence of dual cones, and interestingly, a higher number of dual cones was observed in the three diabetic retinas compared to non-diabetic ones [23]. (2) Another study revealed that Ansell’s mole-rats naturally exhibited low levels of T4, likely as an adaptation to the harsh subterranean ecological conditions. This low T4 level helped them to maintain a reduced resting metabolic rate (RMR) in response to the harsh environment [24]. (3) The association between low levels of free THs and microangiopathy may be attributed to the impairment of vascular function, which is characterized by a decrease in the availability of nitric oxide (NO). This vascular dysfunction leads to alterations in renal hemodynamics, glomerular filtration, and disrupted autoregulation [25]. (4) Czarnyojtek showed that patients with TD exhibited elevated levels of C-reactive protein (CRP), which could potentially contribute to the onset and progression of DR [26].

At present, there are limited studies on the incidence of DR in patients with T2DM complicated with subclinical hypothyroidism (SCH). However, a meta-analysis investigating the association between DR and SCH has revealed that SCH was linked to an increased risk of DR. Specifically, individuals with SCH had a 2.13 times higher likelihood of developing DR [27]. A recent study further corroborated these findings, demonstrating that patients with SCH experienced greater severity of DR compared to those with normal thyroid function. Additionally, the severity of DR increased significantly with the increase in TSHs [28]. Hence, it is recommended that patients with SCH undergo regular retinal examinations for the early detection and diagnosis of DR. In our study, the subjects were all with normal levels of THs. We also found that TSH levels were higher in both the NPDR and PDR groups compared to the NDR group. After adjusting for age, sex, duration, blood pressure, blood glucose, and other factors, the high level of TSHs within the normal range was an independent risk factor for DR (OR = 1.94,95%CI 1.35, 2.80). Simultaneously, in this study, we used the ROC curve to evaluate the predictive effect of TSHs on DR. The TSH cut-off point was 2.38uIU/ml.

Multiple studies have indicated that certain oral antidiabetic drugs can impact the level of THs. In an animal study, the effects of metformin on external symptoms and biochemical indicators related to thyroid function were assessed in rats. The findings revealed that metformin administration led to alterations in thyroid function in male rats, characterized by clinical manifestations of hyperthyroidism. The role of metformin in inhibiting TSH levels and increasing FT3 and FT4 levels highlights the importance of clinical awareness regarding the therapeutic intervention of metformin in thyroid-related conditions [29]. Also, another study has shown that sulfonylureas may increase the risk of thyroid cancer and the risk of hypothyroidism and goitre [30]. In this study, the utilization of hypoglycemic drugs was also assessed in patients with DR at various stages. The results indicated no significant statistical difference among the three groups in terms of metformin, sulfonylureas, and other hypoglycemic medications. This finding suggests that the influence of hypoglycemic medications on TH detection was effectively excluded from the study.

TT4RI [31] is a simple index for evaluating the pituitary sensitivity of thyroid hormones, which was first proposed by Jostel et al. It is used to estimate the maximum pituitary TSH reserve by extrapolating the TSH feedback inhibition amount to the standardized unsuppressed TSHs with an FT4 value of 0. TFQI [9] is a percentile-based thyroid feedback index, which was first proposed by Martin Laclaustra’s team. One advantage of utilizing TT4RI is that it maintains stability and avoids generating excessively high values when dealing with abnormal thyroid function. FT3 is primarily generated through the conversion of iodothyronine DIO in peripheral tissues. The ratio of FT3 to FT4, known as FT3/FT4, can indicate the sensitivity of peripheral organs and tissues to fluctuations in FT4 levels [32]. At present, there are limited studies on the correlation between central and peripheral TH sensitivity and the complications of T2DM.

Regarding the correlation between TH sensitivity and T2DM, recent studies have shown that higher TH resistance indices are associated with obesity, metabolic syndrome, diabetes, and diabetes-related mortality. Hence, it is proposed that TH resistance can serve as an indicator of the energy balance in individuals with T2DM. These resistance indices may offer valuable insights into monitoring the energy balance of T2DM patients [9]. In our study, we examined the relationship between the levels of TFQI, TT4QI, and TSHI, which serve as indicators of central TH sensitivity, and the occurrence of DR in euthyroid patients with T2DM. Our results of univariate regression analysis showed that high levels of TFQI, TSHI, and TT4RI were independent risk factors for DR after the adjustment of age, sex, diabetes duration, blood glucose, and blood pressure, which indicate that the central TH sensitivity may be a risk factor for DR in T2DM patients. Also, the results suggest that after adjusting for age, sex, duration, blood pressure, blood glucose, and other factors, central TH sensitivity is also a risk factor for PDR.

At the same time, in this study, we also analyzed the correlation between the sensitivity of peripheral organs and tissues to changes in THs and DR. The results showed that FT3/FT4 levels in the NPDR and PDR groups were significantly lower compared to the NDR group. In contrast to our research findings, a recent cross-sectional study reported no statistically significant association between the sensitivity of TH indices and the risk of DR in euthyroid patients with T2DM after adjusting for covariates in the logistic regression model [33]. However, the larger sample size utilized in our study may better reveal the correlation between TH sensitivity and DR. The HPT axis plays an important role in retinal development, photoreceptor cell differentiation, cone opsin expression, and regulation of photoreceptor cell survival. Within this axis, the TH receptor-β (TR-β) serves as a crucial regulatory factor for the sensory development of both the cochlea and the retina. On the contrary, in the hypothalamus and pituitary gland, TR-β mediates the inhibitory effect of THs on the expression of thyroid-stimulating hormone-releasing hormones and TSHs, thereby controlling the HPT axis. This may serve as the physiological and pathological basis for the correlation between the TH sensitivity index and retinopathy [34].

There are some limitations in this study. Firstly, due to the cross-sectional design, further research is required to investigate whether modulating thyroid function can potentially improve the severity and prognosis of DR. Secondly, the subjects of this study consisted exclusively of hospitalized patients, potentially limiting the generalizability of the findings to T2DM patients in outpatient settings, specifically concerning the correlation between THs and DR. Additionally, the single measurement of DR examination and TH levels due to the limited duration of hospitalization may introduce deviation, so it is necessary to further study the samples on a large scale in the future. Finally, as this study was conducted retrospectively, certain functional alterations, such as color vision defects, which can be observed in diabetes without evident retinopathy, were not examined. In future studies, it is essential to incorporate some functional parameters among patients with T2DM, as these indicators are also believed to be associated with thyroid homeostasis.

## Conclusion

In summary, this study observed that patients with NPDR and PDR exhibited significantly lower sensitivity levels of central and peripheral thyroid hormones compared to NDR patients, despite having normal thyroid function. After adjusting for factors such as age, sex, duration, blood pressure, blood lipid, and blood glucose, the decreased sensitivity of central and peripheral thyroid hormones was an independent risk factor for the occurrence of DR. These findings have important implications for predicting the risk of development and progression of DR, which may lay the groundwork for conducting large-scale cohort studies. Furthermore, these provide valuable insights for developing strategies related to the prevention, treatment, and management of DR in patients with T2DM.

## Data Availability

The datasets generated during the current study are available from the corresponding author on reasonable request.

## References

[1] Cheng YJ, Kanaya AM, Araneta MRG, et al. Prevalence of diabetes by race and ethnicity in the United States, 2011–2016. JAMA. 2019;322(24):1–11. doi: 10.1001/jama.2019.19365. PMC6990660 31860047

[2] Chatterjee S, Khunti K, Davies MJ. Type 2 diabetes. Lancet. 2017;389(10085):2239–2251. doi: 10.1016/S0140-6736(17)30058-2. 28190580

[3] Wong TY, Cheung CMG, Larsen M, et al. Diabetic retinopathy. Nat Rev Dis Primers. 2016;2(1):16012. doi: 10.1038/nrdp.2016.12. 27159554

[4] Chaker L, Ligthart S, Korevaar TI, et al. Thyroid function and risk of type 2 diabetes: a population-based prospective cohort study. BMC Med. 2016;14(1):150. doi: 10.1186/s12916-016-0693-4. 27686165 PMC5043536

[5] Zou J, Li Z, Tian F, et al. Association between normal thyroid hormones and diabetic retinopathy in patients with type 2 diabetes. Biomed Res Int. 2020;2020:8161797. doi: 10.1155/2020/8161797. 32104706 PMC7040386

[6] Qi Q, Zhang QM, Li CJ, et al. Association of thyroid-stimulating hormone levels with microvascular complications in type 2 diabetes patients. Med Sci Monit. 2017;23:2715–2720. doi: 10.12659/msm.902006. 28578377 PMC5467710

[7] Yang JK, Liu W, Shi J, et al. An association between subclinical hypothyroidism and sight-threatening diabetic retinopathy in type 2 diabetic patients. Diabetes Care. 2010;33(5):1018–1020. doi: 10.2337/dc09-1784. 20150298 PMC2858165

[8] van der Spek AH, Fliers E, Boelen A. The classic pathways of thyroid hormone metabolism. Mol Cell Endocrinol. 2017;458:29–38. doi: 10.1016/j.mce.2017.01.025. 28109953

[9] Laclaustra M, Moreno-Franco B, Lou-Bonafonte JM, et al. Impaired sensitivity to thyroid hormones is associated with diabetes and metabolic syndrome. Diabetes Care. 2019;42(2):303–310. doi: 10.2337/dc18-1410. 30552134

[10] Nie X, Ma X, Xu Y, et al. Increased serum adipocyte fatty acid-binding protein levels are associated with decreased sensitivity to thyroid hormones in the euthyroid population. Thyroid. 2020;30(12):1718–1723. doi: 10.1089/thy.2020.0011. 32394790

[11] World Health Organization. Definition, diagnosis and classification of diabetes mellitus and its complications. Report of a WHO consultation. Part 1: diagnosis and classification of diabetes mellitus. Geneva: WHO 1999. p. 4–7.

[12] Kim B. Thyroid hormone as a determinant of energy expenditure and the basal metabolic rate. Thyroid. 2008;18(2):141–144. ]doi: 10.1089/thy.2007.0266. 18279014

[13] Salvatore D, Simonides WS, Dentice M, et al. Thyroid hormones and skeletal muscle–new insights and potential implications. Nat Rev Endocrinol. 2014;10(4):206–214. doi: 10.1038/nrendo.2013.238. 24322650 PMC4037849

[14] Jun JE, Jee JH, Bae JC, et al. Association between changes in thyroid hormones incident type 2 diabetes: a seven-year longitudinal study. Thyroid. 2017;27(1):29–38. doi: 10.1089/thy.2016.0171. 27809684

[15] Lambadiari V, Mitrou P, Maratou E, et al. Thyroid hormones are positively associated with insulin resistance early in the development of type 2 diabetes. Endocrine. 2011;39(1):28–32. doi: 10.1007/s12020-010-9408-3. 21072691

[16] Stefanowicz-Rutkowska MM, Baranowska-Jurkun A, Matuszewski W, et al. Thyroid dysfunction in patients with diabetic retinopathy. Endokrynologia Polska. 2020;71(2):176–183. doi: 10.5603/EP.a2020.0013. 32396211

[17] Oda T, Taneichi H, Takahashi K, et al. Positive association of free triiodothyronine with pancreatic β‐cell function in people with prediabetes. Diabet Med. 2015;32(2):213–219. doi: 10.1111/dme.12589. 25255697

[18] Kong X, Wang J, Gao G, et al. Association between free thyroxine levels and di abetic retinopathy in euthyroid patients with type 2 diabetes mellitus. Endocr Res. 2020;45(2):111–118. doi: 10.1080/07435800.2019.1690504. 31773995

[19] Hu Y, Hu Z, Tang W, et al. Association of thyroid hormone levels with microvascular complications in euthyroid type 2 diabetes mellitus patients. Diabetes Metab Syndr Obes. 2022;15:2467–2477. doi: 10.2147/DMSO.S354872. 35982763 PMC9380826

[20] Kocaturk T, Ergin K, Cesur G, et al. The effect of methimazole-induced postnatal hypothyroidism on the retinal maturation and on the sirtuin 2 level. Cutan Ocul Toxicol. 2016;35(1):36–40. doi: 10.3109/15569527.2015.1007509. 25758293

[21] Ittermann T, Dörr M, Völzke H, et al. High serum thyrotropin levels are associated with retinal arteriolar narrowing in the general population. Thyroid. 2014;24(10):1473–1478. doi: 10.1089/thy.2014.0190. 25156414

[22] Énzsöly A, Hajdú RI, Turóczi Z, et al. The predictive role of thyroid hormone levels for early diabetic retinal changes in experimental rat and human diabetes. Invest Ophthalmol Vis Sci. 2021;62(6):20. doi: 10.1167/iovs.62.6.20. PMC8142702 34010957

[23] Wu J, Yue S, Geng J, et al. Relationship between diabetic retinopathy and subclinical hypothyroidism: a meta-analysis. Sci Rep. 2015;5:12212. doi: 10.1038/srep12212. 26193340 PMC4507396

[24] Poznyak A, Grechko AV, Poggio P, et al. The diabetes Mellitus-Atherosclerosis connection: the role of lipid and glucose metabolism and chronic inflammation. Int J Mol Sci. 2020;21(5):1835. doi: 10.3390/ijms21051835. 32155866 PMC7084712

[25] Hu X, Liu Y, Wang C, et al. Metformin affects thyroid function in male rats. Oncotarget. 2017;8(64):107589–107595. doi: 10.18632/oncotarget.22536. 29296189 PMC5746091

[26] Henning Y, Mladěnková N, Burda H, et al. Retinal S-opsin dominance in ansell’s mole-rats (fukomys anselli) is a consequence of naturally low serum thyroxine. Sci Rep. 2018;8(1):4337. doi: 10.1038/s41598-018-22705-y. 29531249 PMC5847620

[27] Zhao W, Li X, Liu X, et al. Thyroid function in patients with type 2 diabetes mellitus and diabetic nephropathy: a single center study. J Thyroid Res. 2018;2018:9507028. doi: 10.1155/2018/9507028. 30631416 PMC6304540

[28] Czarnywojtek A, Owecki M, Zgorzalewicz-Stachowiak M, et al. The role of serum C-reactive protein measured by high-sensitive method in thyroid disease. Arch Immunol Ther Exp (Warsz). 2014;62(6):501–509. doi: 10.1007/s00005-014-0282-1. 24794233 PMC4244578

[29] Huston-Paterson HH, Mao Y, Kim J, et al. Thyroid cancer risk is not increased in diabetic patients. Thyroid. 2023. doi: 10.1089/thy.2023.0241.

[30] Jostel A, Ryder WDJ, Shalet SM. The use of thyroid function tests in the diagnosis of hypopituitarism: definition and evaluation of TSH index. Clin Endocrinol. 2009;71(4):529–534. doi: 10.1111/j.1365-2265.2009.03534.x. 19226261

[31] Park SY, Park SE, Jung SW, et al. Free FT3/FT4 rather than thyrotropin is more associated with metabolic parameters in healthy euthyroid adult subjects. Clin Endocrinol. 2017;87(1):87–96. doi: 10.1111/cen.13345. 28374508

[32] Yang J, Ding W, Wang H, et al. Association between sensitivity to thyroid hormone indices and diabetic retinopathy in euthyroid patients with type 2 diabetes mellitus. Diabetes Metab Syndr Obes. 2023;16:535–545. doi: 10.2147/DMSO.S399910. 36874555 PMC9984276

[33] El-Eshmawy MM, Shahin M. Thyroid and eye: where they meet in clinical practice. Endocr Metab Immune Disord Drug Targets. 2020;20(1):39–49. doi: 10.2174/1871530319666190618120107. 31237221

[34] Shuying Li, Kun Qiao, Yao Jiang,et al. Disruptive effects of two organotin pesticides on the thyroid signaling pathway in Xenopus laevis during metamorphosis. Sci Total Environ. 2019;697:134140. doi: 10.1016/j.scitotenv.2019.134140 31476497

